# Diagnostic test accuracy of anti-glycopeptidolipid-core IgA antibodies for *Mycobacterium avium complex* pulmonary disease: systematic review and meta-analysis

**DOI:** 10.1038/srep29325

**Published:** 2016-07-04

**Authors:** Yuji Shibata, Nobuyuki Horita, Masaki Yamamoto, Toshinori Tsukahara, Hideyuki Nagakura, Ken Tashiro, Hiroki Watanabe, Kenjiro Nagai, Kentaro Nakashima, Ryota Ushio, Misako Ikeda, Atsuya Narita, Akinori Kanai, Takashi Sato, Takeshi Kaneko

**Affiliations:** 1 Department of Pulmonology, Yokohama City University Graduate School of Medicine, 3-9, Fukuura, Kanazawa, Yokohama, Japan

## Abstract

Currently, an anti-glycopeptidolipid (GPL)-core IgA antibody assay kit for diagnosing *Mycobacterium avium* complex (MAC) is commercially available. We conducted this systematic review and meta-analysis to reveal the precise diagnostic accuracy of anti-GPL-core IgA antibodies for MAC pulmonary disease (MAC-PD). We systematically searched reports that could provide data for both sensitivity and specificity by anti-GPL-core IgA antibody for clinically diagnosed MAC-PD. Diagnostic test accuracy was estimated using the bivariate model. Of the 257 articles that we had found through primary search, we finally included 16 reports consisted of 1098 reference positive subjects and 2270 reference negative subjects. The diagnostic odds ratio was 24.8 (95% CI 11.6–52.8, I^2^ = 5.5%) and the area under the hierarchical summary receiver operating characteristic curves was 0.873 (95% CI 0.837–0.913). With a cutoff value of 0.7 U/mL, the summary estimates of sensitivity and specificity were 0.696 (95% CI 0.621–0.761) and 0.906 (95% CI 0.836–0.951), respectively. The positive and negative likelihood ratios were 7.4 (95% CI 4.1–13.8) and 0.34 (95% CI 0.26–0.43), respectively. The demanding clinical diagnostic criteria may be a cause of false positive of the index test. The index test had good overall diagnostic accuracy and was useful to ruling in MAC-PD with the cutoff value.

*Mycobacterium* (*M*.) *avium* complex (MAC) is the most common type of non-tuberculosis mycobacterium and often causes chronic pulmonary disease in both immunocompromised and immunocompetent persons. MAC infection usually presents as pulmonary disease but can involve other organs^1^. Because the worldwide prevalence of MAC has greatly increased during the past three decades^2^, nowadays, clinicians often need to diagnose MAC pulmonary disease (PD) in daily practice. Even though we have limited evidence of the effectiveness of antibiotics treatment for MAC, rapid and accurate diagnosis is crucial for deciding treatment and follow-up plans^3^,^4^.

The microbiological diagnostic criteria of MAC-PD in the American Thoracic Society and Infectious Diseases Society of America 2007 Statement (ATS/IDSA 2007 statement) requires two culture-positive sputum samples or one culture positive bronchial lavage^4^. However, the diagnosis of MAC by culture has some limitations. First, the sputum culture is not sufficiently sensitive especially for solitary-nodule cases. Second, the mycobacterium culture needs a long incubation time. Third, we should consider the risk of contamination because MAC is a ubiquitous bacterium found in the environment.

Glycopeptidolipid (GPL) core, which is composed of fatty acid, three amino acids, and rhamnose, is a common major cell wall component of the *M. avium* and *M. intracellulare*^5^. The majority of the other mycobacterium species do not have this core component except for rapidly growing mycobacteria (RGM), namely *M. abscessus, M. chelonae, and M. fortuitum*^5^. After comparing diagnostic power of IgG, IgA, and IgM antibodies for anti-GPL-core, IgA antibody was selected as a candidate target of enzyme immune assay to diagnose MAC-PD^6^. Currently, an anti-GPL-core IgA antibody assay kit for MAC is commercially available^5^. Some studies have reported the diagnostic test accuracy of the test^7^,^8^,^9^,^10^,^11^,^12^,^13^,^14^,^15^,^16^,^17^,^18^,^19^,^20^,^21^,^22^. However, there was considerable inconsistency in the results of these studies. We conducted this systematic review and meta-analysis to reveal the precise diagnostic accuracy of anti-GPL-core IgA antibodies for MAC-PD.

## Methods

### Study registration

The protocol of the current systematic review was drafted following the Preferred Reporting Items for Systematic Reviews and Meta- Analyses (PRISMA) statement and the Cochrane Handbook for Diagnostic Test Accuracy Reviews^23^,^24^. It has been registered with the international prospective register of systematic reviews (PROSPERO) as number CRD42016035449^25^.

### Eligibility criteria

#### Type of studies and participants

We included both one-gate and two-gate studies^24^.

A so-called cohort study that included only MAC-PD-suspected cases had one gate. A so-called case-control study that included both MAC-PD cases and non-MAC-PD cases had two gates. In addition, a study that included MAC-PD-suspected cases and MAC-PD cases and a study that included both MAC-PD-suspected cases and non-MAC-PD had two gates. The two-gate studies had a high risk of bias concerning patient selection^26^.

#### Index test

We evaluated anti-GPL-core IgA antibody for MAC-PD as an index test. Along with a commercialized kit, Capilia MAC Ab ELISA (TAUNS, Shizuoka, Japan), in-house assays were also allowed. Sensitivity and specificity were evaluated using a cutoff value of 0.7 U/mL^27^.

#### Reference test

Reference diagnoses based on ATS/IDSA 2007 statement and American Thoracic Society 1997 statement were preferred^4^,^28^. However, other clinical diagnoses were also accepted.

#### Outcomes

After extracting the number of subjects with true positives/false negatives/false positives/true negatives, we composed a two-by-two contingency table. Overall diagnostic test accuracy was assessed using a diagnostic odds ratio (DOR), and the area under the hierarchical summary receiver operating characteristic (HSROC) curves (AUC). The summary estimates of sensitivity and specificity, positive likelihood ratio (PLR), and negative likelihood ratio (NLR) were also assessed. Positive predictive value and negative predictive value were obtained across the pretest probability ranging from 0% to 100%.

### Literature search strategy

In the electronic search, we systematically searched Pubmed, EMBASE, the Cochrane Library, and Web of Science on February 18^th^, 2016. We used the following search formula for Pubmed without limitation: ((Mycobacterium avium complex) OR (Mycobacterium avium-complex) OR MAC OR MAC-PD OR (non-tuberculosis Mycobacterium)) AND (glycopeptidolipid OR anti-glycopeptidolipid OR anti-glycopeptidolipid-core OR GPL OR anti-GPL OR Capilia OR tauns OR (EIA kit) OR (ELISA kit) OR (enzyme immunoassay kit)) AND (sensitivity OR specificity OR “predictive value” OR “likelihood” OR “true positive” OR “true negative” OR “false positive” OR “false negative” OR diagnostic OR diagnosis). We used similar search formulas for EMBASE, the Cochrane Library, and Web of Science (Supplementary Text 1).

We hand-searched published reviews and included original studies.

### Study selection

Two investigators (YS, NH) independently screened the candidate reports by reading only the title and abstract. Then, the two investigators independently scrutinized the full text of reports that had not been excluded by at least one investigator. Duplicate use of the same data was carefully assessed. The final inclusion was determined by discussion between the two investigators.

### Data extraction

The two investigators (YS, NH) independently extracted the data from the original studies. These data were cross-checked. If necessary, an investigator (NH) tried to contact the author of original reports by e-mail.

### Quality assessment for bias and applicability

The two investigators (YS, NH) independently scored the seven domains of the Revised Tool for the Quality Assessment of Diagnostic Accuracy Studies (QUADAS-2)^26^. When a study might include definite-MAC patients under treatment, the study was scored for high risk of bias concerning patient selection. The final score was determined after discussion between the two investigators. A study that had no domain with a high risk of bias and no domain with high applicability concerns was regarded as a high-quality study.

### Statistical analysis and quantitative synthesis

#### Data synthesis and interpretation

We obtained the DOR using a DerSimonian-Laird random-model and the AUC using Holling’s proportional hazard model. Based on Jones’ criteria^29^, we interpreted AUC >0.97, 0.93–0.96, 0.75–0.92, and 0.5–0.75 as “excellent,” “very good,” “good,” and “reasonable,” respectively. We obtained a paired forest plot, HSROC curve, and the summary estimate of the sensitivity and the specificity using the bivariate model^24^. PLR and NLR were calculated from the summary estimates of sensitivity and specificity. Following Grimes’s criteria^30^, we interpreted PLR in the range of 2–5, 5–10, and >10 as representing small, moderate, and large increases of probability when the index test was positive. We also interpreted the NLR in the range of 0.2–0.5, 0.2–0.1, and <0.1 as representing small, moderate, and large decreases of probability when the index test was negative. The PPV and the NPV that were calculated from the summary estimate of sensitivity and specificity were presented as variables depending on the pretest probability of MAC-PD ranging from 0% to 100%.

#### Sensitivity analysis

As part of the sensitivity analysis, subgroup analysis was conducted focusing on reports that evaluated Capilia MAC Ab ELISA (cutoff: 0.70 U/mL), high-quality reports, and reports evaluating RA cases^5^,^26^.

#### Heterogeneity

We evaluated the heterogeneity using I^2^ statistics. I^2^ < 40% is usually considered “not important” heterogeneity^31^.

#### Software

We used commands of the statistics software R as follows: “madauni” command for DOR, “phm” command for AUC, and “reitsma” command for the HSROC curve and the summary estimates of sensitivity and specificity^32^,^33^.

## Results

### Study search and study characteristics

**Figure 1:**
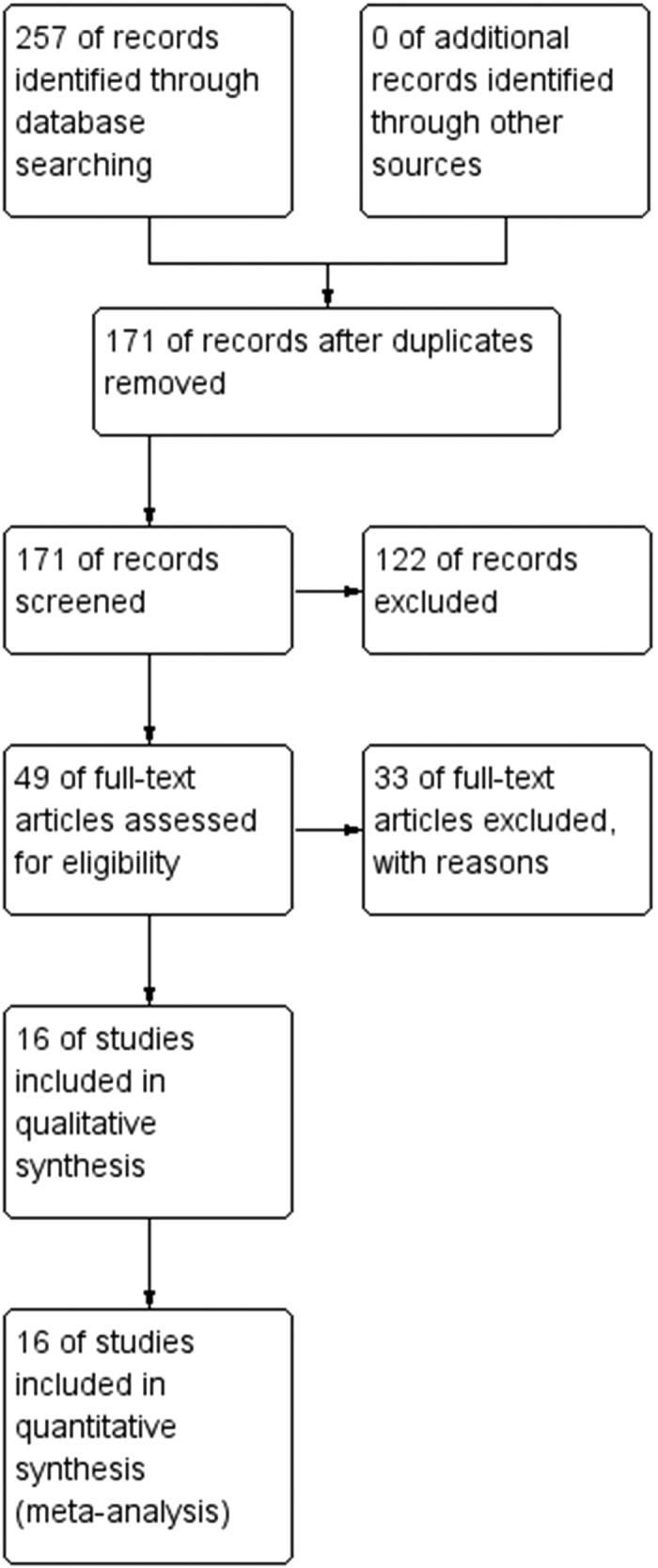
Preferred Reporting Items for Systematic Reviews and Meta- Analyses flow chart for study search.

Of the 257 articles that we had found through primary search, 86, 122, and 33 were excluded through removal of duplication, screening, and full-article reading, respectively (Fig. 1). Notably, Kitada *et al*. reported a number of reports from a hospital, some of which were excluded due to a possible overlap of included subjects. Our hand search found no eligible article.

We finally included 16 reports, comprising 10 full-length articles and six conference abstracts, all of which were written in English (Table 1)^7^,^8^,^9^,^10^,^11^,^12^,^13^,^14^,^15^,^16^,^17^,^18^,^19^,^20^,^21^,^22^. We obtained non-published data concerning four reports through e-mail communication with the authors^16^,^17^,^18^,^19^. Among the 16 reports, 13 were from Japan and one each was from of Korea, the USA, and Taiwan. The Capilia kit was used in 14 studies. The cutoff value of 0.70 U/mL was used in 15 studies. The diagnostic criteria of ATS/IDSA 2007 were used in 10 studies. Four studies used the one-gate cohort approach, all of which were regarded as high-quality studies (Supplementary Figure 1). Three studies included only subjects with rheumatoid arthritis (RA).

**Figure 2:**
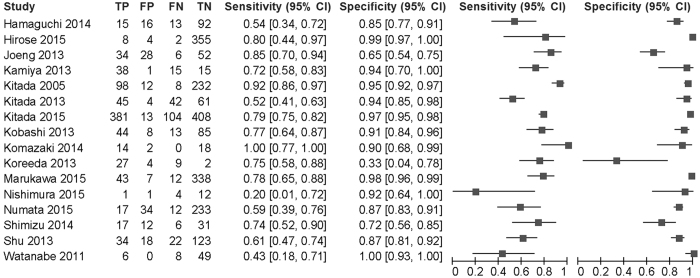
The paired forest plot by anti-glycopeptidolipid-core IgA antibody for *M. avium* complex pulmonary disease. TP: true positive. FP: false positive. FN: false negative. TN: true negative.

The number of participants in each study ranged from 18 to 906 with a median of 143. The total number of subjects was 3368. This total consisted of 1098 reference positive subjects and 2270 reference negative subjects. Across the 16 studies, the sensitivity ranged from 0.20 to 1 with a median of 0.75, and the specificity ranged from 0.33 to 1 with a median of 0.92 (Fig. 2).

### Overall diagnostic accuracy

**Figure 3:**
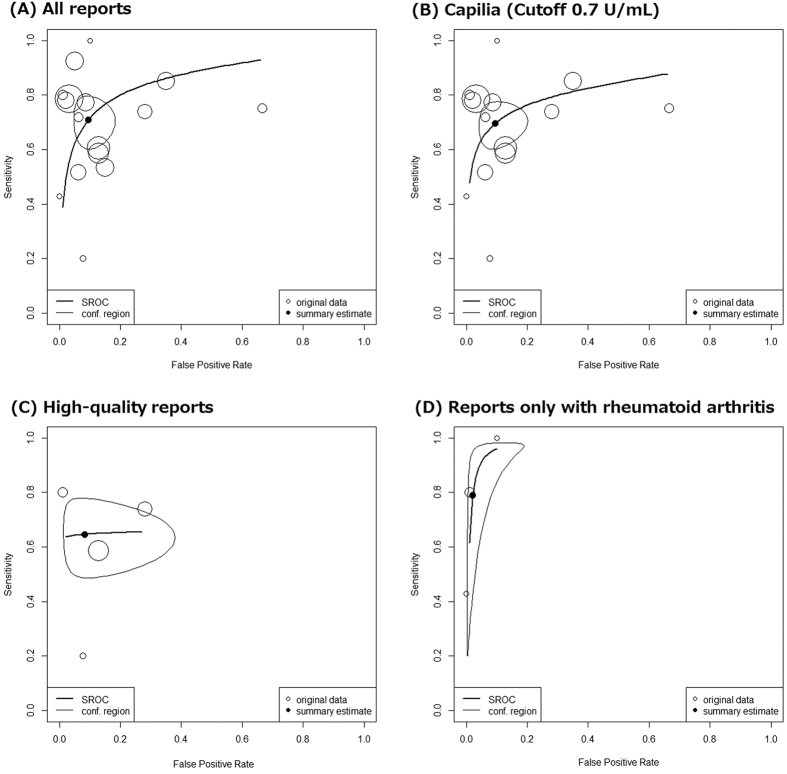
Hierarchical summary receiver operating characteristic curves.

Using data from all 16 studies consisting of 1098 MAC-PD subjects and 2270 non-MAC-PD subjects, DOR was 24.8 (95% confidence interval (95% CI) 11.6–52.8, I^2^ = 5.5%) and AUC was 0.873 (95% CI 0.837–0.913) (Table 2 and Fig. 3A). In accordance with a criterion of Jones *et al*., AUC of 0.873 was categorized as “good”.

As sensitivity analyses, we used two subgroups. When focusing on 14 studies that used the Capilia kit and the cutoff value of 0.7 U/mL, the DOR was 23.1 (95% CI 10.7–50.1, I^2^ = 7.2%) and the AUC was 0.874 (95% CI 0.838–0.913) (Table 2 and Fig. 3B). Four high-quality studies yielded the DOR of 17.4 (95% CI 3.5–87.1, I^2^ = 31.9%) and the AUC of 0.853 (95% CI 66.5–1.000). (Table 2 and Fig. 3C). Based on three reports that included only RA cases, the DOR was 200.1 (95% CI 53.0–754.9 I^2^ = 0%) and the AUC was 0.946 (0.898–0.999) (Table 2 and Fig. 3D).

### Sensitivity and specificity

The summary estimates of sensitivity and specificity were calculated from 14 studies that used the Capilia kit with the cutoff value of 0.7 U/mL. The summary estimates of sensitivity and specificity were 0.696 (95% CI 0.621–0.761) and 0.906 (95% CI 0.836–0.951), respectively. (Table 2 and Fig. 3B)

According to a sensitivity analysis using four high-quality studies, the summary estimates of sensitivity and specificity were 0.646 (95% CI 0.519–0.756) and 0.918 (95% CI 0.706–0.981), respectively (Table 2 and Fig. 3C). The summary estimates sensitivity of 0.790 (95% CI 0.301–0.971) and specificity of 0.979 (95% CI 0.873–0.997) were obtained from three reports including only RA cases.

### Positive and negative likelihood ratios

**Figure 4:**
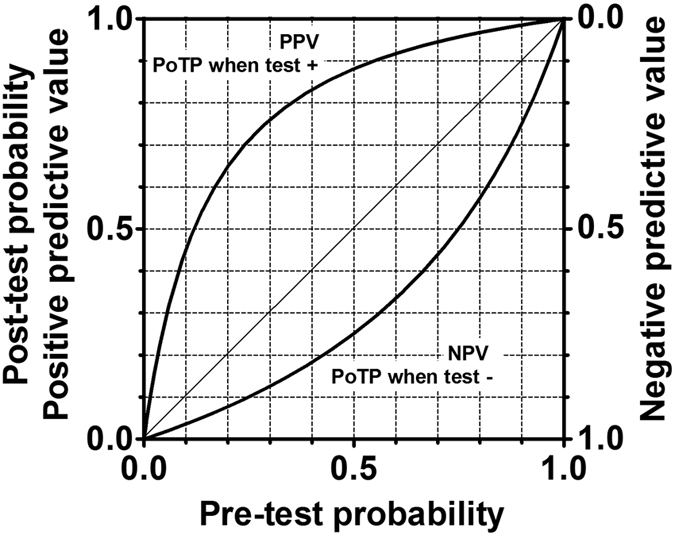
Predictive values. PPV: positive predictive values. NPV: negative predictive values. PoTP: post-test probability.

Based on the summary estimate of sensitivity and specificity calculated from 14 reports using the Capilia kit with the cutoff value of 0.7 U/mL, PLR and NLR were 7.4 (95% CI 4.1–13.8) and 0.34 (95% CI 0.26–0.43). According to Grimes’ criteria, a positive index test moderately increases the probability of MAC-PC, while a negative index test suggests a small decrease of the probability (Table 2 and Fig. 4).

### Positive and negative predictive values

We calculated PPV and NPV from the summary estimate of sensitivity and specificity calculated from 14 reports using the Capilia kit with the cutoff value of 0.7 U/mL, i.e. a sensitivity of 0.696 and a specificity of 0.906 (Fig. 4). This figure indicates that the positive test has a stronger impact than the negative test.

### Positive rate in non-MAC-PD subjects

Some included studies provided data concerning the positive rate in non-MAC-PD subjects. The anti-GPL-core IgA antibody was positive in 27–100% of RGM cases, while the test was negative in 93–100% of pulmonary-healthy controls, 90–100% of *M. tuberculosis* cases, and 97–100% of non-MAC non-RGM NTM-PD cases (Table 3). The pooled positive rate for rapid growing *Mycobacterium* was 0.64 (95% CI 0.27–1.00) (Supplementary Figure 2).

## Discussion

To the best of our knowledge, the current report is the first systematic review and meta-analysis evaluating the diagnostic test accuracy of anti-GPL-core IgA antibody for MAC-PD. We think the results from our study are robust for various reasons. First, we included a sufficient number of studies and subjects. Second, we used the recently recommended hierarchical meta-analysis approach. Third, across all analyses, the observed heterogeneities were not important (<40%). Fourth, a sensitivity analysis revealed consistent results.

Approximately 8% of reference negative subjects had a positive result as regards the anti-GPL-core antibody assessed with the Capilia kit. There are two plausible explanations for a subject with the positive anti-GPL-core IgA antibody assay and the negative clinical diagnosis, usually by ATS/IDSA 2007 statement. First, all MAC, *M. abcessus* and other RGM commonly have GPL antigen in their cell walls^5^. Thus, the anti-GPL-core antibody assay intrinsically has cross-reactivity for MAC and RGM. This is a serious pitfall when interpreting the results of the anti-GPL-core antibody assay because different antibiotic regimens should be prescribed to treat MAC and RGM^3^,^4^. When the anti-GPL IgA antibody is positive, we should carefully consider the possibility of RGM infection, especially *M. abcessus*^9^. While pulmonary imaging studies for MAC-PD commonly present multiple discrete pulmonary nodules^34^, common CT findings of RGM-PD are bilateral bronchiectasis, bronchiolitis, and upper lobe cavities^35^. Second, the universally used diagnostic criteria by ATS/IDSA 2007 statement, which is simpler than the previous American Thoracic Society 1997 statement, are still demanding^4^. The ATS/IDSA 2007 diagnostic criteria overlook some of true MAC-PD population. Although the culture isolation of the microbe has been the gold standard diagnostic method of infectious disease, the sero-diagnosis by anti-GPL-core IgA antibody, for example combining one MAC culture positive plus anti-GPL-core IgA antibody positive, can be used for diagnostic criteria^5^.

Kitada *et al*. proposed a cutoff value for the anti-GPL-core IgA antibody of 0.7 U/mL^27^. This is because this cutoff can provide high specificity, though it is not sensitive. The summary estimate of sensitivity was 69% in our analysis (Table 2), which means that almost a third of MAC-PD cases were overlooked by the anti-GPL-core IgA antibody assay. The serum enzyme immunoassay requires a sufficient quantity of targeted antibody to identify the MAC-infected individual. However, non-extensive pulmonary lesions, especially solitary nodules, and an immunocompromised state, namely an HIV-infected state and use of immunosuppressants, lead to a low serum antibody level^13^.

We need to discuss the limitations of our study. First, most of the included studies had a high risk of bias due to the two-gate study design. Because the prevalence of MAC is not high, it is difficult to recruit a large number of patients with MAC in a cohort study. Nonetheless, we believe the results of our analysis are reliable. This is because the high-quality studies based on sensitivity subgroup analysis that included only one-gate studies yielded consistent results (Table 2 and Fig. 3C). Second, most of the included studies were from Japan and only three were from other countries (Table 1). To reconfirm the diagnostic test accuracy of the sero-diagnosis for MAC, Kitada *et al*. evaluated 152 subjects in the USA and revealed that the sensitivity and specificity were 70.1% and 93.9%, respectively^12^. Third, subgroup analysis focusing on RA cases revealed higher accuracy than results from overall reports (Table 2 and Fig. 3D). However, the analysis was not conclusive due to the lack of a plausible biological explanation and the limited number of included reports and subjects. Fourth, it is not likely that through checkup including BALF was done for every non-MAC persons to completely deny the possibility of MAC due to ethical regulation.

In conclusion, we conducted the first systematic review and meta-analysis concerning the diagnostic test accuracy of anti-GPL-core IgA antibody for MAC-PD. According to our analysis using data of 16 reports and 3368 subjects. The DOR was 24.8 and the AUC was 0.873. The summary estimates of sensitivity and specificity were 0.691 and 0.919, respectively. Considering the demanding clinical diagnostic criteria of the ATS/IDSA 2007 statement, the true specificity of the anti-GPL-core IgA antibody may be higher. A positive index test moderately increases the probability of MAC-PC, while a negative index test suggests a small decrease of the probability. This test is useful to ruling in MAC-PD.

## Additional Information

**How to cite this article**: Shibata, Y. *et al*. Diagnostic test accuracy of anti-glycopeptidolipid-core IgA antibodies for *Mycobacterium avium complex* pulmonary disease: systematic review and meta-analysis. *Sci. Rep*. **6**, 29325; doi: 10.1038/srep29325 (2016).

## Supplementary Material

Supplementary Information

## Figures and Tables

**Table 1:** Characteristics of included studies.

Author (Year)	Country	Gates	Patient background	Report type	Facility	Assay	Cutoff (U/mL)	Reference criteria	Subjects	Quality
Hamaguchi^7^	Japan	2	MAC-PD s/o, MAC-PD	CA	A Red Cross Hp		0.7	ATS	136	Low
Hirose^8^	Japan	1	MAC-PD s/o, screening (all had RA)	FA	A rheumatoid clinic	Capilia	0.7	ATS/IDSA 2007	369	High
Joeng^9^	Korea	2	MAC-PD, MAB-PD, TB, HC	FA	A medical center	Capilia	0.7	ATS/IDSA 2007	120	Low
Kamiya^10^	Japan	2	MAC-PD, non-MAC NTM, TB, LK	CA	A secondary referral Hp	Capilia	0.7	ATS	69	Low
Kitada^11^	Japan	2	MAC-PD, MAC colonization, MKA, TB, HC	FA	A teaching Hp	In-house EIA	0.072	ATS 1997	350	Low
Kitada^12^	USA	2	MAC-PD s/o, HC	FA	A research Hp	Capilia	0.7	ATS/IDSA 2007	152	Low
Kitada^13^	Japan	2	MAC-PD, MKA-PD, TB, HC	FA	A teaching Hp	Capilia	0.7	ATS/IDSA 2007	906	Low
Kobashi^14^	Japan	2	MAC-PD, MAC-PD s/o, TB, non-MAC NTM, PD, HC	FA	Uni and affiliated Hps	Capilia	0.7	ATS/IDSA 2007	150	Low
Komazaki^15^	Japan	2	MAC-PD, MAC r/o (all had RA)	FA	A uni Hp	Capilia	0.7	ATS/IDSA 2007	34	Low
Koreeda^16^	Japan	2	MAC-PD, MAB-PD, other NTM	CA	Uni and affiliated Hps	Capilia	0.7		42	Low
Marukawa^17^	Japan	2	MAC-PD, MACctm, TB, other PD, HC	CA	A medical center	Capilia	0.7		400	Low
Nishimura^18^	Japan	1	MAC-PD s/o, asymptomatic	CA	A teaching Hp	Capilia	0.7	ATS/IDSA 2007	18	High
Numata^19^	Japan	1	MAC-PD s/o	FA	A uni Hp	Capilia	0.7	ATS/IDSA 2007	296	High
Shimizu^20^	Japan	1	MAC-PD s/o	FA	A Red Cross Hp	Capilia	0.7	ATS/IDSA 2007	66	High
Shu^21^	Taiwan	2	MAC-PD, MACctm, RGM, MKA, TB, HC	FA	A uni Hp	Capilia	0.7	ATS/IDSA 2007	197	Low
Watanabe^22^	Japan	2	MAC-PD, non-MAC NTM, abnormal CT, normal chest (All had RA)	FA	Uni and tertiary referral Hps	Capilia	0.7	ATS	63	Low

<Gates> A so-called cohort study that included only MAC-PD-suspected cases had one gate. A so-called case-control study that included both MAC-PD cases and non-MAC-PD cases had two gates. <Patients background> MAC: *M. avium* complex. MACctm: MAC contamination. MAC s/o: suspected diagnosis of MAC. MAB: *M. abcessus*. MKA: *M. kansasii*. RGM: rapidly growing mycobacteria. TB: *M. tuberculosis*. NTM: non-tuberculosis mycobacterium. RA: Rheumatoid arthritis. LK: lung cancer. PD: pulmonary disease. HC: healthy control. <Report type> FA: Full-length article. CA: Conference abstract. All were written in English. <Facility> Uni: university. Hp: hospital. <Assay> EIA: enzyme immunoassay. <Reference criteria> ATS: the American Thoracic Society. IDSA: the Infectious Diseases Society of America. <Quality> A study that had no domain with high risk of bias and no domain with high applicability concerns was regarded as a high-quality study.

**Table 2:** Summary of diagnostic accuracy by anti-glycopeptidolipid-core IgA antibody assay for *M. avium* complex-pulmonary disease (MAC-PD).

	All reports	Capilia (cutoff value 0.7 U/mL)	High-quality reports	Rheumatoid arthritis
Studies	16	14	4	3
MAC-PD reference positive	1098	964	67	38
MAC-PD reference negative	2270	1918	682	432
Diagnostic odds ratio	24.8 (11.6–52.8) I^2^ = 5.5%	23.1 (10.7–50.1) I^2^ = 7.2%	17.4 (3.5–87.1) I^2^ = 31.9%	200.1 (53.0–754.9) I^2^ = 0%
AUC	0.873 (0.837–0.913)	0.874 (0.838–0.913)	0.853 (0.665–1.000)	0.946 (0.898–0.999)
Sensitivity	Not available	0.696 (0.621–0.761)	0.646 (0.519–0.756)	0.790 (0.301–0.971)
Specificity	Not available	0.906 (0.836–0.951)	0.918 (0.706–0.981)	0.979 (0.873–0.997)
Positive likelihood ratio	Not available	7.4 (4.1–13.8)	7.9 (2.2–33.7)	37.6 (4.8–253.5)
Negative likelihood ratio	Not available	0.34 (0.26–0.43)	0.39 (0.27–0.57)	0.21 (0.03–0.73)

Brackets indicate 95% confidence interval. High quality reports: A study that had no domain with high risk of bias and no domain with high applicability concerns was regarded as a high-quality study. AUC: area under hierarchical summary receiver operating characteristics curve. Main outcomes concerning diagnostic accuracy are written in *italics*. The others are results from sensitivity analyses.

**Table 3:** Positive rate in non-MAC subjects.

	Pulmonary-healthy control	*M. tuberculosis*	RGM	Non-MAC non-RGM NTM	Non-MAC NTM collectively
Joeng^9^	0/20 (0%)	0/20 (0%)	28/40 (70%)		28/40 (70%)
Kamiya^10^		0/7 (0%)			1/4 (25%)
Kitada^13^	4/126 (3%)	4/77 (5%)		3/30 (10%)	
Kitada^12^	3/52 (6%)				
Kobashi^14^	0/20 (0%)	0/18 (0%)			0/9 (0%)
Komazaki^15^	0/45 (0%)				
Koreeda^16^			4/4 (100%)	0/2 (0%)	4/6 (67%)
Numata^19^		0/12 (0%)			1/5 (20%)
Shu^21^	3/42 (7%)	5/48 (10%)	7/26 (27%)	0/14 (0%)	7/40 (18%)
Watanabe^22^	0/30 (0%)				0/3 (0%)

MAC: *M. avium* complex. RGM: rapidly growing mycobacterium including *M. abcessus*. NTM: non-tuberculosis mycobacterium.
