# Supplementary material for: Diagnostic test accuracy of anti-glycopeptidolipid-core IgA antibodies for Mycobacterium avium complex pulmonary disease: systematic review and meta-analysis
Source: Sci Rep. 2016 Jul 4;6:29325. doi: 10.1038/srep29325 (PMC4931691; doi:10.1038/srep29325)
Supplement: Supplementary Information [file srep29325-s1.doc]

**Diagnostic test accuracy of anti-glycopeptidolipid-core IgA antibodies for Mycobacterium avium complex pulmonary disease: systematic review and meta-analysis.**

**Authors**

^1)^Yuji Shibata, ^1)^Nobuyuki Horita, ^1)^Masaki Yamamoto, ^1)^Toshinori Tsukahara, ^1)^Hideyuki Nagakura, ^1)^Ken Tashiro, ^1)^Hiroki Watanabe, ^1)^Kenjiro Nagai, ^1)^Kentaro Nakashima, ^1)^Ryota Ushio, ^1)^Misako Ikeda, ^1)^Atsuya Narita, ^1)^Akinori Kanai, ^1)^Takashi Sato, ^1)^Takeshi Kaneko.

**Supplementary File**

**Supplementary Text 1. Electrical search formulas.**

Web of Science Core Collection advanced search without limitation:

TS=((mycobacterium avium complex) OR (mycobacterium avium-complex) OR MAC OR MAC-PD OR (non-tuberculosis Mycobacterium)) AND TS=(glycopeptidolipid OR anti-glycopeptidolipid OR anti-glycopeptidolipid-core OR GPL OR anti-GPL OR Capilia OR tauns OR(EIA kit) OR (ELISA kit) OR (enzyme immunoassay kit)) AND TS=(sensitivity OR specificity OR "predictive value" OR "likelihood" OR "true positive" OR "true negative" OR "false positive" OR "false negative" OR diagnostic OR diagnosis).

Cochrane advanced search Wiley Online Library title/abstract/keyword search without limitation:

((mycobacterium avium complex) OR (mycobacterium avium-complex) OR MAC OR MAC-PD OR (non-tuberculosis Mycobacterium)) AND (glycopeptidolipid OR anti-glycopeptidolipid OR anti-glycopeptidolipid-core OR GPL OR anti-GPL OR Capilia OR tauns OR(EIA kit) OR (ELISA kit) OR (enzyme immunoassay kit))

Embase advanced search without limitation:

((mycobacterium AND avium AND complex) OR ('mycobacterium'/exp OR mycobacterium AND 'avium complex') OR mac OR 'mac pd' OR ('non tuberculosis' AND ('mycobacterium'/exp OR mycobacterium))) AND (glycopeptidolipid OR 'anti glycopeptidolipid' OR 'anti glycopeptidolipid core' OR gpl OR 'anti gpl' OR capilia OR tauns OR ('eia'/exp OR eia AND kit) OR ('elisa'/exp OR elisa AND kit) OR ('enzyme'/exp OR enzyme AND ('immunoassay'/exp OR immunoassay) AND kit)) AND (sensitivity OR specificity OR 'predictive value'/exp OR 'predictive value' OR 'likelihood' OR 'true positive' OR 'true negative' OR 'false positive' OR 'false negative' OR diagnostic OR 'diagnosis'/exp OR diagnosis)

**Supplemental Figure 1. A Revised Tool for the Quality Assessment of Diagnostic Accuracy Studies Risk of bias and applicability concerns graph.**

**
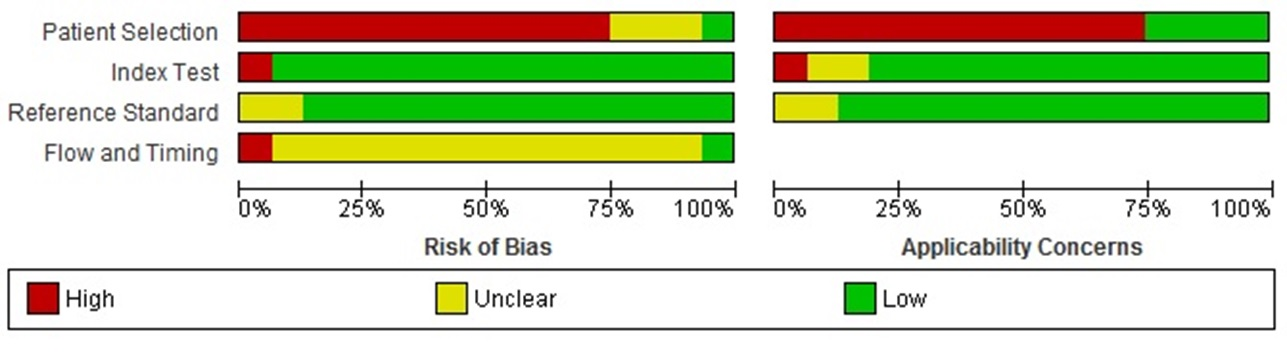
**


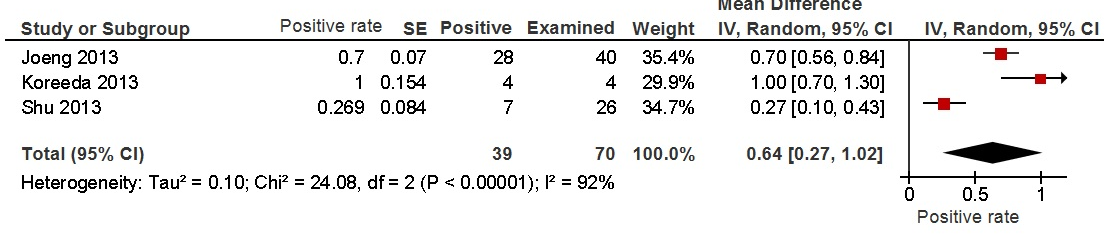
**Supplemental Figure 2. The pooled positive rate of anti-GPL-core IgA antibody for RGM: rapidly growing mycobacterium including *M. abcessus*.**

SE was estimated using Agresti-Coull method.
